# Supplementary material for: Molecular Characterization and Demographic Study on Infectious Bursal Disease Virus in Faisalabad District
Source: PLoS One. 2021 Aug 16;16(8):e0254605. doi: 10.1371/journal.pone.0254605 (PMC8366999; doi:10.1371/journal.pone.0254605)
Supplement: Attachment — Submitted filename: PONE-D-21-08105_reviewer.pdf [file pone.0254605.s001.pdf]

PLOS ONE

Molecular characterization and demographic study on infectious bursal disease virus in district Faisalabad

--Manuscript Draft--

**Manuscript Number:**	PONE-D-21-08105
**Article Type:**	Research Article
**Full Title:**	Molecular characterization and demographic study on infectious bursal disease virus in district Faisalabad
**Short Title:**	demographic study on infectious bursal disease virus
**Corresponding Author:**	Sanaullah Sajid, M.Phil/PhD<br>UAF: University of Agriculture Faisalabad<br>Faisalabad, Not Applicable PAKISTAN
**Keywords:**	poultry; IBDV; VP2 gene; Hypervariable region; Phylogenetic tree
**Abstract:**	The re-emergence of virulent strains of infectious bursal disease (IBD) virus leads to significant economic losses to the poultry industry in Pakistan in the last few years. This disease causes the infection of bursa, which leads to major immune losses. Total 30 number of samples from five IBD outbreaks during the period of 2019-20 were collected from different areas of district Faisalabad and assayed by targeting the IBD virus VP2 region through RT-PCR. Among all the disease outbreaks, almost 80% birds were found positive for IBD virus. Tissues were collected from the diseased birds, histopathological examination of IBDV revealed the severe lymphocytic depletion, infiltration of inflammatory cells and necrosis of the bursa of Fabricius. Positive samples were subjected to further isolation and molecular characterization of IBDV. The Pakistan IBDV genes was subjected to DNA sequencing to determine the virus nucleotide sequences. The sequences of 100 serotype-I IBDVs showing nearest homology were compared and identified with study sequence. The phylogenetic tree for nucleotide sequences was constructed using neighbor-joining method in MEGA-6 with reference strains. representative strains. The VP2 segment reassortment of IBDVs carrying segment A were identified as one important type of circulating strains in Pakistan. The data discovered the molecular features of the Pakistan IBDV strains playing a role in the evolution of new strains of the virus, will contribute to the vaccine selection and effective prevention of the disease.
**Order of Authors:**	Sanaullah Sajid, M.Phil/PhD
**Additional Information:**	
**Question**	**Response**
**Financial Disclosure**<br><br>Enter a financial disclosure statement that describes the sources of funding for the work included in this submission. Review the submission guidelines for detailed requirements. View published research articles from *PLOS ONE* for specific examples.<br><br>This statement is required for submission and **will appear in the published article** if the submission is accepted. Please make sure it is accurate.	NO

Powered by Editorial Manager® and ProduXion Manager® from Aries Systems Corporation

**Unfunded studies**<br>Enter: *The author(s) received no specific funding for this work.*<br><br>**Funded studies**<br>Enter a statement with the following details:<br>• Initials of the authors who received each award<br>• Grant numbers awarded to each author<br>• The full name of each funder<br>• URL of each funder website<br>• Did the sponsors or funders play any role in the study design, data collection and analysis, decision to publish, or preparation of the manuscript?<br>• **NO** - Include this sentence at the end of your statement: *The funders had no role in study design, data collection and analysis, decision to publish, or preparation of the manuscript.*<br>• **YES** - Specify the role(s) played.<br><br>* typeset	
**Competing Interests**<br><br>Use the instructions below to enter a competing interest statement for this submission. On behalf of all authors, disclose any competing interests that could be perceived to bias this work—acknowledging all financial support and any other relevant financial or non-financial competing interests.<br><br>This statement is required for submission and **will appear in the published article** if the submission is accepted. Please make sure it is accurate and that any funding sources listed in your Funding Information later in the submission form are also declared in your Financial Disclosure statement.<br><br>View published research articles from *PLOS ONE* for specific examples.	NO authors have competing interests

**NO authors have competing interests**<br><br>Enter: *The authors have declared that no competing interests exist.*<br><br>**Authors with competing interests**<br><br>Enter competing interest details beginning with this statement:<br><br>*I have read the journal's policy and the authors of this manuscript have the following competing interests: [insert competing interests here]*<br><br>* typeset	
**Ethics Statement**<br><br>Enter an ethics statement for this submission. This statement is required if the study involved:<br><br>• Human participants<br>• Human specimens or tissue<br>• Vertebrate animals or cephalopods<br>• Vertebrate embryos or tissues<br>• Field research<br><br>Write "N/A" if the submission does not require an ethics statement.<br><br>General guidance is provided below. Consult the submission guidelines for detailed instructions. **Make sure that all information entered here is included in the Methods section of the manuscript.**	No animal is sacrificed in this study. But the study is approved by the Institutional Biosafety Committee, University of Agriculture Faisalabad Pakistan.

Powered by Editorial Manager® and ProduXion Manager® from Aries Systems Corporation

**Format for specific study types**<br><br>**Human Subject Research (involving human participants and/or tissue)**<br>• Give the name of the institutional review board or ethics committee that approved the study<br>• Include the approval number and/or a statement indicating approval of this research<br>• Indicate the form of consent obtained (written/oral) or the reason that consent was not obtained (e.g. the data were analyzed anonymously)<br><br>**Animal Research (involving vertebrate animals, embryos or tissues)**<br>• Provide the name of the Institutional Animal Care and Use Committee (IACUC) or other relevant ethics board that reviewed the study protocol, and indicate whether they approved this research or granted a formal waiver of ethical approval<br>• Include an approval number if one was obtained<br>• If the study involved *non-human primates*, add *additional details* about animal welfare and steps taken to ameliorate suffering<br>• If anesthesia, euthanasia, or any kind of animal sacrifice is part of the study, include briefly which substances and/or methods were applied<br><br>**Field Research**<br><br>Include the following details if this study involves the collection of plant, animal, or other materials from a natural setting:<br>• Field permit number<br>• Name of the institution or relevant body that granted permission	
**Data Availability**<br><br>Authors are required to make all data underlying the findings described fully available, without restriction, and from the time of publication. PLOS allows rare exceptions to address legal and ethical concerns. See the PLOS Data Policy and FAQ for detailed information.	Yes - all data are fully available without restriction

A Data Availability Statement describing where the data can be found is required at submission. Your answers to this question constitute the Data Availability Statement and **will be published in the article**, if accepted.<br><br>**Important:** Stating 'data available on request from the author' is not sufficient. If your data are only available upon request, select 'No' for the first question and explain your exceptional situation in the text box.<br><br>Do the authors confirm that all data underlying the findings described in their manuscript are fully available without restriction?	
**Describe where the data may be found in full sentences. If you are copying our sample text, replace any instances of XXX with the appropriate details.**<br><br>• If the data are **held or will be held in a public repository**, include URLs, accession numbers or DOIs. If this information will only be available after acceptance, indicate this by ticking the box below. For example: *All XXX files are available from the XXX database (accession number(s) XXX, XXX.*).<br>• If the data are all contained **within the manuscript and/or Supporting Information files**, enter the following: *All relevant data are within the manuscript and its Supporting Information files.*<br>• If neither of these applies but you are able to provide **details of access elsewhere**, with or without limitations, please do so. For example:<br><br>*Data cannot be shared publicly because of [XXX]. Data are available from the XXX Institutional Data Access / Ethics Committee (contact via XXX) for researchers who meet the criteria for access to confidential data.*<br><br>*The data underlying the results presented in the study are available from (include the name of the third party*	Data are available from sanaullahsajid@gmail.com

and contact information or URL).<br>• This text is appropriate if the data are owned by a third party and authors do not have permission to share the data.<br><br>* typeset	
Additional data availability information:	Tick here if your circumstances are not covered by the questions above and you need the journal's help to make your data available.; Tick here if the URLs/accession numbers/DOIs will be available only after acceptance of the manuscript for publication so that we can ensure their inclusion before publication.

Powered by Editorial Manager® and ProduXion Manager® from Aries Systems Corporation

Click here to access/download;Manuscript;Mol Characterization Final-MM.docx

Molecular characterization and demographic study on infectious bursal disease virus in district Faisalabad

Sanaullah Sajid[1], Sajjad ur Rahman[1*], Mashkoor Mohsin Gilani[1], Zia ud Din Sindhu[2]

[1]Institute of Microbiology, University of Agriculture, Faisalabad
[2]Department of parasitology, University of Agriculture, Faisalabad

Abstract

The re-emergence of virulent strains of infectious bursal disease (IBD) virus leads to significant economic losses to the poultry industry in Pakistan in the last few years. This disease causes the infection of bursa, which leads to major immune losses. Total 30 number of samples from five IBD outbreaks during the period of 2019-20 were collected from different areas of district Faisalabad and assayed by targeting the IBD virus VP2 region through RT-PCR. Among all the disease outbreaks, almost 80% birds were found positive for IBD virus. Tissues were collected from the diseased birds, histopathological examination of IBDV revealed the severe lymphocytic depletion, infiltration of inflammatory cells and necrosis of the bursa of Fabricius. Positive samples were subjected to further isolation and molecular characterization of IBDV. The Pakistan IBDV genes was subjected to DNA sequencing to determine the virus nucleotide sequences. The sequences of 100 serotype-I IBDVs showing nearest homology were compared and identified with study sequence. The phylogenetic tree for nucleotide sequences was constructed using neighbor-joining method in MEGA-6 with reference strains. representative strains. The VP2 segment reassortment of IBDVs carrying segment A were identified as one important type of circulating strains in Pakistan. The data discovered the molecular features of the Pakistan IBDV strains playing a role in the evolution of new strains of the virus, will contribute to the vaccine selection and effective prevention of the disease.

Keywords: Poultry, IBDV, VP2 gene, Hypervariable region, Phylogenetic tree

Introduction

Gumboro is a highly contagious disease of fowl caused by infectious bursal disease virus (IBDV). IBDV belongs to Avibirnaviridae group of Birnaviridae family. IBD virus is non-enveloped dsRNA virus with a genome having 2 segments A and B with a diameter of 55–60 nm. Two serotypes of this virus have been recognized, of which only serotype-I is known to cause disease in birds (Sajid *et al.*, 2020). Serotype-II infects turkeys and several avian species, but no disease has been reported due to it. Segment A is self-cleaved to yield protease and structural proteins pVP2 and VP3. The pVP2 encodes the VP2 as a major capsid protein of the virus. A wide variety of pathotypes of serotype-I exist in nature that have been classified as clinical virulent, subclinical virulent and very virulent groups (Jackwood *et al.,* 2011; Van den Berg *et al.,* 2004). Despite the efforts of vaccination, outbreaks of infectious bursal disease virus (IBDV) are still reported worldwide as a significant immunosuppressive disease of fowl. The reassortment of IBD virus genome, genomic recombination and frequent genetic mutations, potentially alter antigenicity and increase virulence which renders the vaccines least effective. Since virus is resistant to heat inactivation, chemicals and highly contagious so, eradication is not practical on infected farms (Jackwood *et al.*, 2008).

The economic losses due to infectious bursal disease experienced by the poultry industry are not only the result of mortality and morbidity but the dramatic fall in the overall performance of the flock. Bursa of Fabricius is the target organ on IBD virus that replicated in the B-lymphocytes resulting in the humoral suppression due to depletion of B cells and compromised cellular immunity (Vukea *et al.*, 2014). The increased susceptibility to the multitude of opportunistic pathogens is due to irreversible immune suppression in the IBD infected birds that result in the economic impacts on the layer and broiler industry that is estimated to be 3.9 million kilograms of meat per year having $14 million market value (Zachar *et al.*, 2016; Shabbir *et al.*, 2016). IBDV is present in clinical and subclinical forms and birds recovered from virus remains carrier. The virus cannot be inactivated and there is no treatment for this disease but it can be controlled by vaccination and biosecurity (Khan *et al.*, 2017; Hussain *et al.,* 2004). The two major antigenic groups within serotype-I are commonly called classical and variant, but antigenic drift has contributed to the formation of several subtypes within these groups. The antigenic phenotype of IBDV is determined by the hypervariable sequence region of VP2 (hvVP2)

specifically, by amino acids located at the apex of loop structures designated P_{BC}, P_{DE}, P_{FG}, and P_{HI} Even single point mutations in these regions have been found to contribute to antigenic drift in IBDV, which can render currently available IBD vaccines ineffective. The phylogenetic studies suggested the expansion of virus was initiated by an event where an endemic segment A was reasserted with segment B of unknown origin (Mawgod *et al.,* 2014; Metwally *et al.,* 2009).

The goal of our study was to identify IBDV strains that continue to cause disease in commercial chicken flocks. Although pathogenicity is important with regard to severity of the disease and degree of immune suppression, we focused our study on mutations located in the hvVP2.

Materials and Methods

Field Samples

Symptomatic bursa samples were submitted from different commercial farms in district Faisalabad. During the year 2019-2020, 30 bursae were obtained from 5 IBD suspected outbreaks in Faisalabad, with 10-18% mortality observed at these farms (Table 1). The diseased birds were manifested by atrophy and inflammation of bursa and the feaces stained fathers around the vent. The bursae samples were transported to the Molecular Research Laboratory, Institute of Microbiology, University of Agriculture Faisalabad for molecular diagnosis and characterization

Table 1 Field samples collected from flocks with signs of disease

Sample ID	Place and Year	Flock size	Age (days)	Category	Vaccination status	Reported Pathogenicity
IOM-2001	F-2019	5500	30 DO	Broiler	V	Low
IOM-2002	F-2019	6500	22 DO	Broiler	NR	Low
IOM-2003	F-2019	10000	35 DO	Layer	NV	High
IOM-2004	F-2019	10000	28 DO	Broiler	NV	NR
IOM-2005	F-2019	1750	30 DO	Layer	V	Low
IOM-2006	F-2019	5700	49 DO	Layer	V	High
IOM-2007	F-2019	1200	42 DO	Layer	NV	High
IOM-2008	F-2019	20000	42 DO	Layer	NV	NR

IOM-2009	F-2019	1000	29 DO	Broiler	NR	Low
IOM-2010	F-2019	15700	27 DO	Broiler	V	Low
IOM-2011	F-2019	10000	36 DO	Layer	V	NR
IOM-2012	F-2019	1250	28 DO	Broiler	V	Low
IOM-2013	F-2020	5000	27 DO	Broiler	V	NR
IOM-2014	F-2020	5200	36 DO	Broiler	NV	High
IOM-2015	F-2020	6000	30 DO	Broiler	V	Low
IOM-2016	F-2020	1000	NR	NR	NV	High
IOM-2017	F-2020	6500	35 DO	Broiler	V	Low
IOM-2018	F-2020	5500	34 DO	Broiler	NR	Low
IOM-2019	F-2020	1250	28 DO	Broiler	V	Low
IOM-2021	F-2020	5800	NR	NR	V	High
IOM-2022	F-2020	10000	25 DO	Broiler	V	High
IOM-2023	F-2020	1200	20 DO	Broiler	NR	NR
IOM-2024	F-2020	10000	NR	NR	V	Low
IOM-2025	F-2020	30000	28 DO	Broiler	V	Low
IOM-2026	F-2020	500	25 DO	Broiler	V	NR
IOM-2027	F-2020	570	31 DO	Broiler	V	High
IOM-2028	F-2020	900	30 DO	Broiler	V	High
IOM-2029	F-2020	600	NR	NR	V	NR
IOM-2030	F-2020	1000	23 DO	Broiler	V	Low

F, Faisalabad; DO, day old; NR, not reported; V, vaccinated; NV, Non vaccinated.

Histopathology

Histopathology of tissues was performed as described by (Bancroft and Gamble 2008). Briefly, Bursa tissues were fixed in 10% neutral buffered formalin solution and left for 7 days. Washed with water to remove fixative and dehydrated with increasing concentration of ethanol, followed by clearance in xylene. Tissues were embedded in paraffin using steel molds and sectioned to 3µm thickness with a microtome. Using Mayer’s egg albumin as an adhesive material, tissues were mounted on glass slides and dried at 37°C for 24 hours. After incubation of 30 minutes at

45°C, tissues were stained with Haematoxylin and eosin stains (Stevens, 1996; Meulemans, 2000).

Electron Microscopy

A small fraction of virus suspension was taken after trituration of bursa centrifugation at 8000 rpm for 5 minutes. Suspension was treated with 0.05 M Tris-HCl with pH 8.0. After staining with 2% sodium phosphotungstate, virus particles were adsorbed to formvar carbon coated grid. Specimen grid was examined in an electron microscope operating at 100 kV (Vancini *et al.*, 2012).

RNA Isolation

The bursae of Fabricius were homogenized in mortar and pestle with PBS supplemented with Kanamycin (700 µg/ml). Samples were clarified by centrifugation @ 8000 rpm for 5 minutes to eliminate the cell debris. Total RNA was extracted using 200 µl of the supernatant using GeneJet viral purification kit (Thermo Fisher) according to the manufacturer's protocol (Ndashe *et al.*, 2016; Muller *et al.*, 2003).

Quantification of complementary DNA

Complementary DNA was quantified in the analytical laboratory of US-Pak Center for Advanced studies, University of Agriculture Faisalabad using Nano-Drop quantification method. The elution buffer was run as a blank and 1µl of viral cDNA samples were placed to record the readings (Lejal *et al.*, 2000).

RT-PCR

The RT reaction was performed using SuperScript™ reverse transcriptase kit (Invitrogen, USA). Total RNA template was mixed in a 20µl reaction containing 50 ng of random hexamer primer, 10 mM dNTP mix, 4 µl of 5X First-Strand buffer, 1 µl of 0.1 M DDT and 200 units of SuperScript™ RT. The IBDV hypervariable VP2 was detected by using primer pair BG-VP2 described in Table 2. The primer pair BG-VP2 produces a DNA amplicon of 699 base pairs. PCR product was visualized following electrophoresis on a 1.5% agarose gel stained with Ethidium Bromide (Vera *at al.*, 2015; Hassan, 2004).

Table. 2 Primer used for the amplification and sequencing of IBDV

Primers	Sequences	Position	Size

BG-F	5´- CATATGTGCAGCCGATGATTACC-3´	751	699 nt
BG-R	5´- CCTGGAATTCTCAGGGGAGAGTTG -3´	1499	

F, forward; R, reverse; nt, nucleotide

Sequence analysis of hyper variable region of IBDV-VP2 gene

The amplified PCR products of VP2 (hypervariable) region were purified using a PCR purification kit (QIAGEN, USA) according to the manufacturer's protocol. Purified VP2 DNA were subjected to DNA sequencing at Advance Bioscience Internationals to determine the virus nucleotide sequences. The sequences were then deposited in the GenBank/NCBI database and the deduced amino acid and nucleotide sequences of IBD virus were aligned using CLUSTAL-W method and subjected to BLAST searches [https://blast.ncbi.nlm.nih.gov/Blast.cgi] to determine the identity with other strains (Abdel-Alim *et al.,* 2003). The sequences of 100 serotype-I IBDVs showing the nearest homology were compared and identified with study sequence. The phylogenetic tree for nucleotide sequences was constructed using representative strains. The topological accuracy of the phylogenetic tree was estimated by the bootstrap method, with 1000 replicates and inference of phylogenetic relationship was determined by neighbor-joining method in MEGA-X (Tamura *et al.*, 2011; Banda *et al.*, 2003).

Phylogenetic analysis of VP2 hypervariable region

Nucleotide sequence data was analyzed using BioEdit, MegaAlign and Lasergene DNASTAR software's using hypervariable region of VP2 gene. Molecular and phylogenetic analysis was conducted using Mega (version 6.0) software by neighbor-joining method with Kimura three parameter evolutionary model. Phylogenetic analysis relies on the arrangement of VP2 nucleotide sequences covering the hypervariable region at the nucleotide 845-1126. The deduced amino acid sequences from 210 to 391 were investigated for genetic markers. The sequence of isolates incorporated in the table. 3 were grouped into seven Geno-groups. Sequenced data from Asia and other regions were selected for the comprehensive studies to evaluate the genetic information based on neighbor joining method. The deduced amino acid sequence profile in the hydrophilic regions was assessed to explore the mutations at amino acid level.

Table. 4 Description of genetic markets in different Geno-groups

Geno-Group	222 [a]	242 [a]	256 [a]	279 [a]	294 [a]	299 [a]

G-1	A/P/S	V/2	V/I/A	D/N	I/L	N
G-2	T/Q	V	V	N	L	N/S
G-3	A/S	I	I	D/N	I	S/N
G-4	S	V	V	N	L	H/S/N
G-5	T	V	V	N	L	N
G-6	Q	V	K/I	D	L	S
G-7	P	V	V	G	L	S

a Amino acid positions in VP2 region of IDB virus

Results

Histopathological examination

Histopathological examination of the bursae showed characteristic lesions attributed to the IBDV infection. Acute and chronic lesions were identified in 30 infected bursae with fibrin deposition and depletion of lymphoid follicles, necrosis, infiltration of heterophilic inflammatory cells, severe atrophy with the presence of vacuoles in the medullary and cortical region. At day one of infection, the necrosis of lymphocytes resulted in karyorrhectic debris followed by characterized edema, hyperemia and reticuloendothelial cell hyperplasia. The inflammation diminished on the fifth day of infection by removal of debris through phagocytosis and the appearance of cystic cavities in the medullary area of lymphoid follicles.

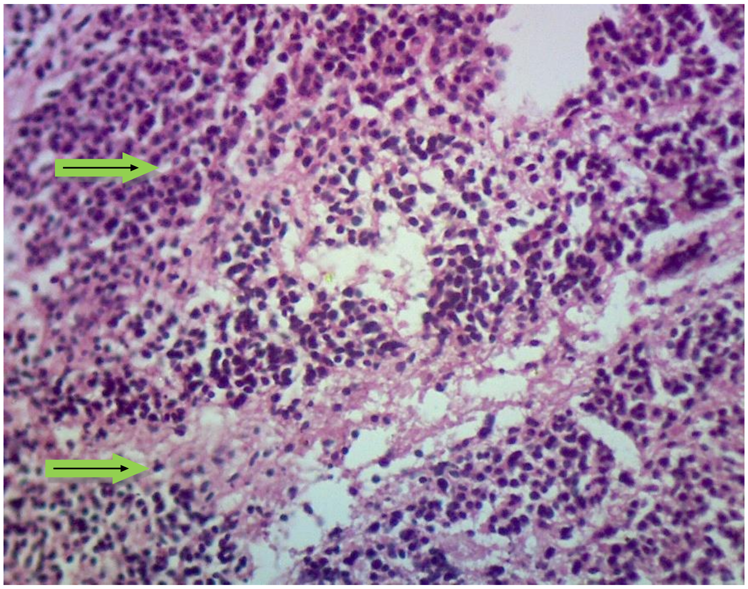

Fig. 1 Shows histopathological section of bursa of Fabricius with infiltration of inflammatory cells, desquamation of surface epithelium at 40X magnification after H&E staining.

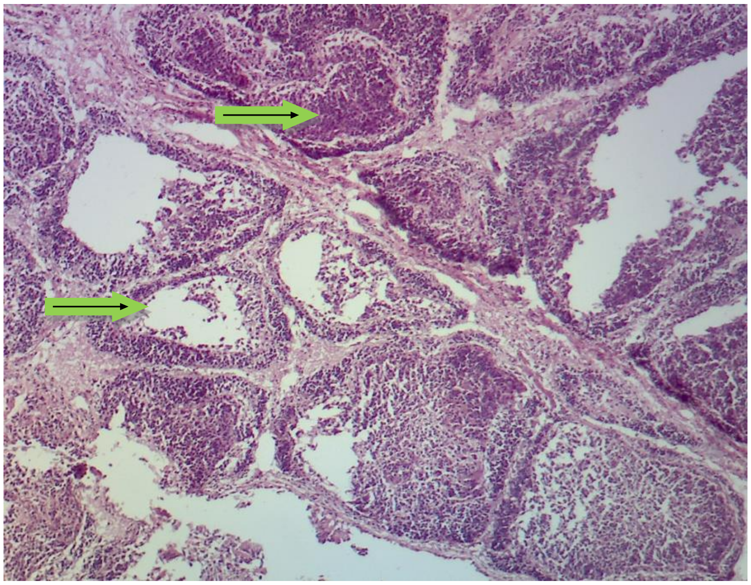

Fig. 2 Shows histopathological section of bursa of Fabricius with lymphocytic depletion with marked atrophy of lymphoid follicles and thickening of interfollicular space by severe fibrosis at 40X magnification after H&E staining.

Electron Microscopy

The icosahedral structure of infectious bursal disease virus was determined from the images obtained by Transmission electron microscope. The reconstruction of IBD virus revealed the icosahedral shape of virus particles with pentameric protrusions on each fivefold vertex. The five to fivefold distance measures 750 angstrom, in which VP2 protein of IBD virus appears to be organized in 260 trimers in fivefold vertices, making the edges and the face of icosahedron.

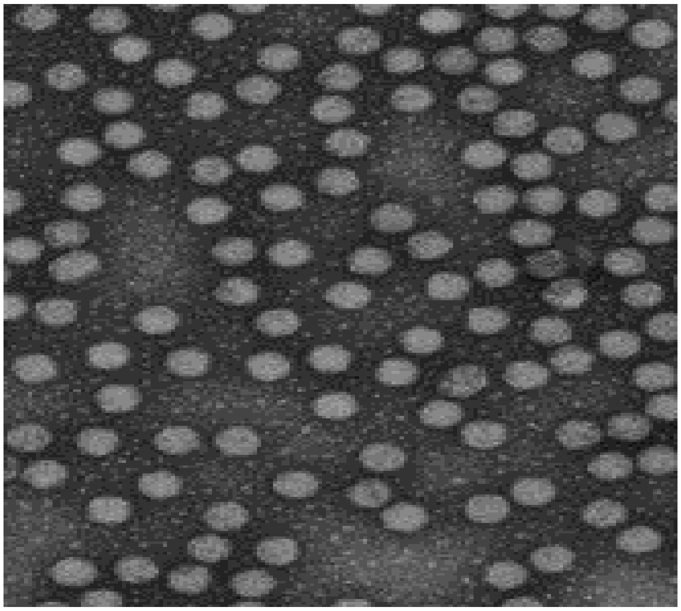

Fig. 3 Transmission electron microscopy of infectious bursal disease virus after negative staining with 2% sodium phosphotungstate

Molecular detection of IBDV VP2 gene

RT-PCR showed that 13% (4/30) bursa tissues were positive for IBDV. A region of 699 nt in segment A (reg VP2) was amplified for sequencing and comparative analysis of isolates. The VP2 region was successfully amplified in this study and added to the analysis. All the four Pakistan strains were submitted to GenBank and the accession numbers are listed in Table 5.

Table. 5 Infectious bursal disease virus strains isolated in the study

No.	Strain	Origin	Collection Date	Host	Accession no.
1	IOMF2019	Faisalabad, Punjab	2019	Layer	MW483681
2	IOMF2019	Faisalabad, Punjab	2019	Broiler	MW483682
3	IOMF2020	Faisalabad, Punjab	2020	Broiler	MW483683
4	IOMF2020	Faisalabad, Punjab	2020	Broiler	MW483684

Sequence analysis of VP2-HVR of Geno-groups

For the detailed analysis, all the sequences were aligned for nucleotide position at 628-1173 and amino acid position at 210-391. Based on constructed phylogenetic tree and similarities, the data displayed into seven genogroups. genogroup 1, comprised of 15 globally reported and diversified isolates in which at amino acid position 222, over 80% of the isolates had Proline (P) and shared S/A in the variable region. Most isolates had Valine (V) at position 242. At position 256, the

positioned amino acid Valine (V) contributed to 88% along with Alanine (A) and Isoleucine (I). At the position 279 in the minor hydrophilic region, the isolates had 12% Asparagine (N) and Aspartate (D) while Leucine (L) was positioned at 294.

In genogroup 2, the amino acids at position 222 in the conserved regions were Threonine (T), 256V, 296L, 242V, 299N and 279N. The reference isolates in this group were limited to non-Asian countries. Previously the classification of the isolates in this group was antigenic variants and confused with classical recombinants of genogroup 5. For the first time these were clearly categorized into a separate group by Jackwood in December 2017 that presented with a universal prevalence of genogroup 3 with typical hallmark similar at 242I, 222A, 256I and 299 S (Serine). Three unique changes at amino acid S317R, D323N and S315T were also reported and the isolates from the Asian border sharing countries had exhibited a heterologous record in the hypervariable regions of the infectious bursal disease virus. In genogroup 4, the amino acid exhibited H/S/Histidine (H) at genetic marker 299 while there were some common amino acids at position 294L, 222S, 279N and 256V. This group having diversifies amino acid profiles but showed similarities to the genogroup 5 and prevalent all over the world. Genogroup 5 shared the same genetic hallmark i.e., 294L, 256V, 222T and 299N. Genogroup 6 only prevailing outside the Asia specially in Russia and Italy, while all the isolates in genogroup 7 shared the same amino acid proline (P) at position 222 and this group is prevalent outside Asia. All the isolates of this group have 100% amino acid similarity.

The phylogenetic studies revealed that the genogroup 1, 3 and 4 shared the genetic information universally, but the other groups were preserved in the specific regions. Classical strains of infectious bursal disease virus were segregated in genogroup 1, while very virulent IBDV strains were grouped in genogroup 3 and these two groups consist of the most existing strains of the virus. The antigen variant strains of virus responsible for most economic losses in the poultry sector belonged to genogroup 2. The distinctive class of IBD virus (dIBDV) previously prevalent outside the Asia was classified in genogroup 4. In this study, one sample matches with the group 4 lineage that needs further investigation. Recombinant IBD viruses classifies in genogroup 5 shared the variant and classical group amino acid profile. The amino acid and genetic profiling of genogroup 6 was reported from Italy and it was later on reported in Russia. The isolates of genogroup 7 were Australian origin and few reported from Russia.

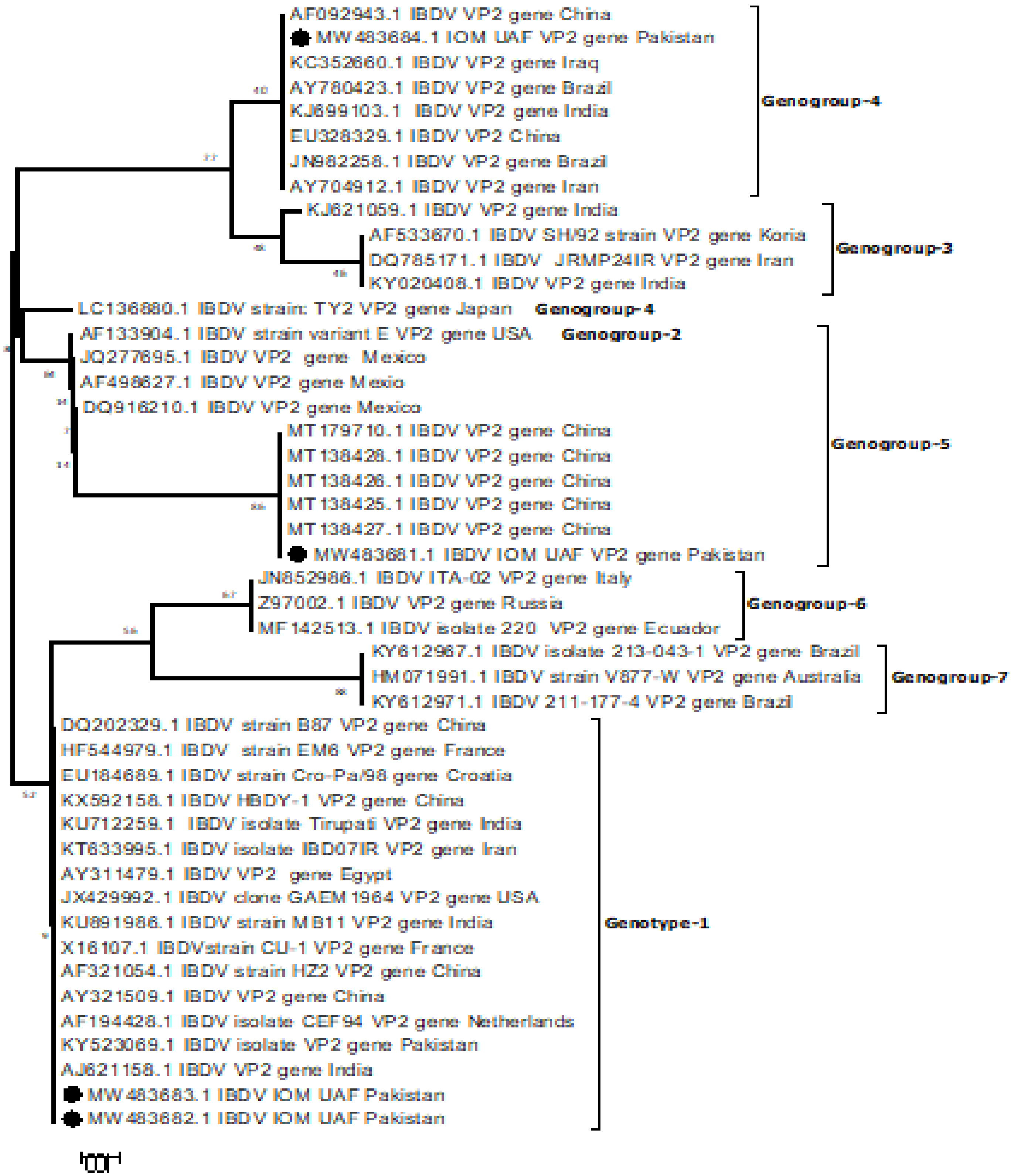

Fig. 5 Phylogenetic consensus tree based on the hypervariable domain of VP2 gene of infectious bursal disease virus, designed by neighbor-joining (NJ) method using Clustal W alignment of the nucleotides created in Mega-6.

Discussion

Among the viral diseases of the poultry birds, infectious bursal disease virus is the second most important disease after Newcastle disease. Due to the immunosuppressive nature of the disease, it continuously adds to the economic losses in the poultry sector due to compromised cellular and humoral responses after the destruction of B cells resulting in the lower feed conversion ratio (FCR). The only hope to control IBD is vaccination however, outbreaks of this disease are also documented in the vaccinated flocks due to the introduction of new virus strains. The possible reason could be the mutation (amino acid changes) that may alter the protection level of the bird. The genogroup 1,2 and 3 accommodated the large population of the IBD viruses while the classical strains fall in the genogroup 1 and the vaccination of the birds is the only way to mitigate the losses caused by the classical IBD while the frequent vaccination at different time intervals in the rearing flock could be a possible cause of mutation in the IBDVs. The quantity, quality and the procedure of vaccination is also questionable. Despite of the variation in the genetic hallmark of Pak-Asian isolates, the position of amino acids at N299 was found conserved in this genogroup.

Genogroup 2 shared the amino acid at position 279N, 299N, 318D, 249K, 256V, 242V and 222T. The most economic losses to the poultry industry reported due to variant strains that lead to misdiagnosis and no pathognomonic lesions resulted in the secondary infections caused by immunosuppression. The genogroup 3 reported to be responsible for the global outbreaks of IBD. The four loop structures in the hypervariable region of VP2 (PDE, PHI, PBC, PFG) were linked to the modification in virulence of the infectious bursal disease virus. In genogroup 4, the amino acid shared the common positions at 296F, 256V, 242V, 279N and 289P. The sequence analysis showed the 92% similarity between the dIBDV and vaccinal strain of IBDV (Winterfield) that predicts the spread of these strains in Asian and non-Asian countries.

Genogroup 5 has common genetic markers with genogroup 2, but this group is restricted to the countries other than Asian. The two substitution at A321P and S317K were observed in major hydrophilic region. At the antigen epitopes of a single IBD virus, the presence of classical and variant sequences provides solid ground to genogroup 5. The IBD virus in the genogroup 6 were found to be circulating in the Italian region but its sequenced data shared the worldwide distribution of the virus. The important gene markers of these isolates included 256K and 253E but the alteration of amino acid at position 222Q, 220H and 321A in major hydrophilic region A

and B classifies these isolates in an independent genogroup 6. In genogroup 7, mostly the Australian isolates were grouped, sharing 100% similarity due to conserved genetic markers and presence in the specific regions.

In the present scenario, trade and the vaccinal strains of the IBD virus might be the reason for the global emergence of new strains with difference in their pathogenicity's. In Pakistan, 16 isolates were submitted in GenBank before this study. In this region, classical strains of IBD virus before 1980s were reported to be the dominant strains of the virus. The mutation at position 222 from proline (P) to Throline (T) considered in the shifting of classical to variant genogroup and the possible reason might be the vaccination pressure due to worldwide use of these strains in for the immunization of rearing flock.

Conclusion

In this study, the IBD virus isolates were found to be present in the genogroup 1, genogroup 4 and genogroup 5. All the three genogroups are prevalent globally while the re-assorted and mutated isolated were confined to the specific regions of the world.

References

Abdel-Alim GA, Awaad MHH and Saif YM (2003). Characterization of Egyptian field strains of infectious bursal disease virus. *Avian diseases 47*(4): 1452-1457.

Banda A, Villegas P, & El-Attrache J (2003). Molecular characterization of infectious bursal disease virus from commercial poultry in the United States and Latin America. *Avian diseases 47*(1): 87-95.

Hassan MK (2004). Very virulent infectious bursal disease virus in Egypt: epidemiology, isolation and immunogenicity of classic vaccine. *Veterinary research communications 28*(4): 347-356.

Hussain I, Rasool MH and Mahmood MS (2004). Production of hyperimmune serum against infectious bursal disease virus in rabbits. *Pak. Vet. J 24*: 179-183.

Jackwood DJ, Sommer-Wagner SE, Crossley BM, Stoute ST, Woolcock PR, & Charlton BR (2011). Identification and pathogenicity of a natural reassortant between a very virulent serotype 1 infectious bursal disease virus (IBDV) and a serotype 2 IBDV. *Virology 420* (2): 98-105.

Jackwood, D. J., Sreedevi, B., LeFever, L. J., & Sommer-Wagner, S. E. (2008). Studies on naturally occurring infectious bursal disease viruses suggest that a single amino acid substitution at position 253 in VP2 increases pathogenicity. *Virology, 377*(1), 110-116.

Khan RSA, Sajid S, Habib M, Ali W, Shah MSUD and Sarfraz M (2017). History of Gumboro (infectious bursal disease) in Pakistan. *Saudi Pharmaceutical Journal 25*(4): 453-459.

Lejal N, Da Costa B, Huet JC and Delmas B (2000). Role of Ser-652 and Lys-692 in the protease activity of infectious bursal disease virus VP4 and identification of its substrate cleavage sites. *Journal of General Virology 81*(4): 983-992.

Mawgod SA, Arafa AS and Hussein HA (2014). Molecular genotyping of the infectious bursal disease virus (IBDV) isolated from Broiler Flocks in Egypt. *International Journal of Veterinary Science and Medicine 2*(1): 46-52.

Metwally AM, Sabry MZ, Samy AM, Omar MM, Yousif AA and Reda IM (2003). Direct detection of variant infectious bursal disease virus in vaccinated Egyptian broiler flocks using antigen-capture ELISA. *Veterinary Medical Journal Giza 51*(1): 105-119.

Metwally AM, Yousif AA, Shaheed IB, Mohammed WA, Samy AM and Reda IM (2009). Re-emergence of very virulent IBDV in Egypt. *International Journal of Virology 5*(1): 1-17.

Meulemans G (2000). Infectious bursal disease (Gumboro disease). *Rev. sci. tech. Off. int. Epiz, 19*(2): 527-543.

Müller H, Islam MR and Raue R (2003). Research on infectious bursal disease—the past, the present and the future. *Veterinary microbiology 97*(1-2): 153-165.

Ndashe K, Simulundu E, Hang'ombe BM, Moonga L, Ogawa H, Takada A, and Mweene AS (2016). Molecular characterization of infectious bursal disease viruses detected in vaccinated commercial broiler flocks in Lusaka, Zambia. *Archives of virology, 161*(3), 513-519.

Sajid, S., ur Rahman, S., Nayab, S., Khan, I. U., & Javeed, A. (2020). 71. Infectious bursal disease virus cloning andstructural protein (VP2) expression in Escherichia coli. *Pure and Applied Biology (PAB), 9*(1), 743-749.

Shabbir MZ, Ali M, Abbas M, Chaudhry UN and Munir M, 2016. Molecular characterization of infectious bursal disease viruses from Pakistan. *Archives of virology, 161*(7), 2001-2006.

Stevens A (1996). The haematoxylin and eosin. *Theory and practice of histological techniques* 99-112.

Tamura K, Peterson D, Peterson N, Stecher G, Nei M and Kumar S (2011). MEGA5: molecular evolutionary genetics analysis using maximum likelihood, evolutionary distance, and maximum parsimony methods. *Molecular biology and evolution 28*(10): 2731-2739.

Vera F, Craig MI, Olivera V, Rojas F, König G, Pereda A and Vagnozzi A. (2015). Molecular characterization of infectious bursal disease virus (IBDV) isolated in Argentina indicates a regional lineage. *Archives of virology*, *160*(8), 1909-1921.

Vancini, R., Paredes, A., Ribeiro, M., Blackburn, K., Ferreira, D., Kononchik, J. P., ... & Brown, D. (2012). Espirito Santo virus: a new birnavirus that replicates in insect cells. *Journal of virology*, *86*(5), 2390-2399.

Van den Berg TP, Morales D, Eterradossi N, Rivallan G, Toquin D, Raue R and Zheng HJ (2004). Assessment of genetic, antigenic and pathotypic criteria for the characterization of IBDV strains. *Avian Pathology 33*(5): 470-476.

Vukea PR, Willows-Munro S, Horner RF and Coetzer TH (2014). Phylogenetic analysis of the polyprotein coding region of an infectious South African bursal disease virus (IBDV) strain. *Infection, Genetics and Evolution 21*: 279-286.

Zachar, T., Popowich, S., Goodhope, B., Knezacek, T., Ojkic, D., Willson, P., ... & Gomis, S. (2016). A 5-year study of the incidence and economic impact of variant infectious bursal disease viruses on broiler production in Saskatchewan, Canada. *Canadian journal of veterinary research*, *80*(4), 255-261.
