# Supplementary material for: Molecular Characterization and Demographic Study on Infectious Bursal Disease Virus in Faisalabad District
Source: PLoS One. 2021 Aug 16;16(8):e0254605. doi: 10.1371/journal.pone.0254605 (PMC8366999; doi:10.1371/journal.pone.0254605)
Supplement: Attachment — Submitted filename: Response to Reviewers.docx [file pone.0254605.s002.docx]

**Response to Reviewers**

| **Reviewers’ Comments** | **Explanation** |
| --- | --- |
| **1.** Revise this sentence in simple way as it looks complexing for readers. Also make it short. | Revised |
| **2.** Cite a fresh reference for this section. | (Naveed *et al.,* 2018). |
| **3.** which tool was used to design the primers. | LaserGene software |
| **4.** You didst mention the similarity index of your results with previous ones. In figure, also no similarity index is available. Revise the figure of phylogenetic analysis. | Mentioned |
| **5.** how you have discussed your results with previous one. No comparison found. Revise it. | References added for comparison |
